# The Governance of UK Dairy Antibiotic Use: Industry-Led Policy in Action

**DOI:** 10.3389/fvets.2020.00557

**Published:** 2020-09-04

**Authors:** Stephanie Begemann, Francine Watkins, Ine Van Hoyweghen, Roberto Vivancos, Robert Christley, Elizabeth Perkins

**Affiliations:** ^1^Knowledge, Technology and Innovation Group, University of Wageningen, Wageningen, Netherlands; ^2^NIHR HPRU in Emerging and Zoonotic Infections, Liverpool, United Kingdom; ^3^Department of Public Health, Institute of Population Health, University of Liverpool, Liverpool, United Kingdom; ^4^Life Sciences & Society Lab, Centre of Sociological Research, KU Leuven, Leuven, Belgium; ^5^Field Service, Public Health England, Liverpool, United Kingdom; ^6^NIHR HPRU in Gastrointestinal Infections, Liverpool, United Kingdom; ^7^Institute of Infection, Veterinary and Ecological Sciences, University of Liverpool, Liverpool, United Kingdom; ^8^Health Services Research, Institute of Psychology Health and Society, University of Liverpool, Liverpool, United Kingdom

**Keywords:** agriculture, antimicrobial resistance, governance, antibiotic policies and practices, matters of concern, actor-network theory

## Abstract

This article analyses the progress made in the UK with regard to tackling antibiotic “misuse and overuse” in food-producing animals. Moving beyond statistical realities, the paper examines how the UK's industry-led policy approach is shaping practice. Using a multi-sited ethnography situated in Actor Network Theory and Callon's sociology of markets, the UK dairy supply chain policies and practices were studied. Findings reveal that dairy industry policies only partially address the complex network of people, animals, and the environment in which dairy antibiotics circulate. Antibiotic “misuse and overuse” in agriculture is far from a behavioural matter, with solely farmers and veterinarians to blame. Instead, antibiotic use in food animals is embedded in complex economic networks that constrain radical changes in dairy husbandry management and antibiotic use on farms. More attention toward the *needs* of the dairy supply chain actors and wider environmental considerations is essential to reduce the dairy sector's dependency on antibiotics and support transition toward responsible farming in the UK.

## Introduction

Antimicrobial resistance (AMR) is one of the most complex public health challenges of the twenty first century. As a consequence, human, and animal antibiotic (AB) use has been internationally problematised with urgent areas for action identified (1). The central issue, with regard to both humans and animals, is *how* to reduce AB-use. Since ABs are used by farmers and veterinary surgeons, international and national policy narratives documents have focussed on the “misuse and overuse” of ABs by these particular actors (1–4). By framing the use of AB-use in food producing animals as “excessive,” the problem has become calculable and amenable to intervention (2). As a result, the quantification of AB sales and use has become central to national and international discussions: metrics are used by policymakers to inform policies and evaluate the impact of these policies.

**Figure 1:**
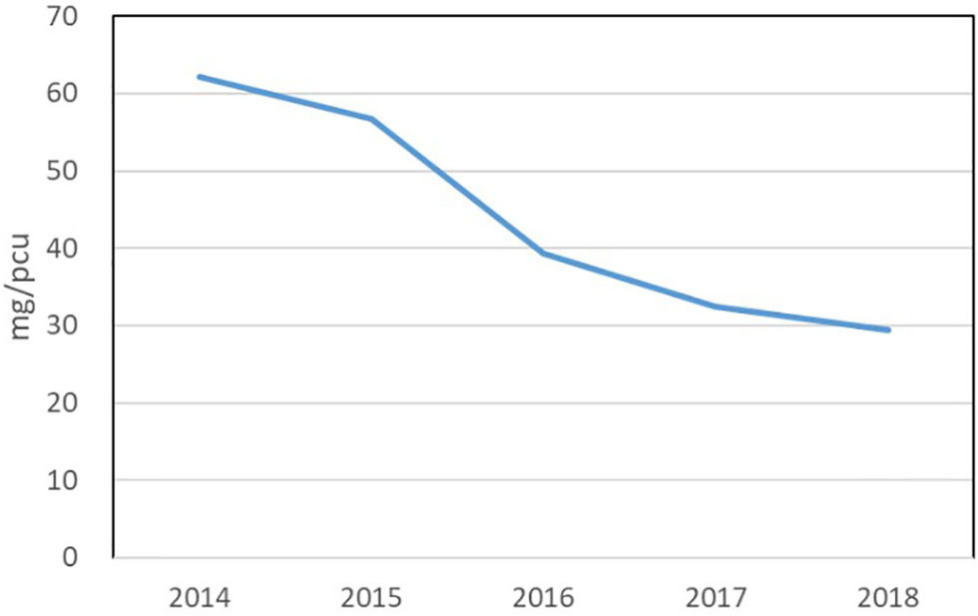
The UK's veterinary antimicrobial sales in mg/PUC 2014–2018 (UK-VARSS 2019).

In the UK, responsible AB-use in its food supply chains is industry-led. In this governance model, the UK government has set AB reduction targets but has made the agricultural industry responsible to design and implement AB policies that will help to achieve those targets. Embedded within the UK's industry-led policy models is a belief that technocratic interventions (AB surveillance systems, AB guidelines, knowledge transfer instruments) promote responsible AB-use and can drive farmer and veterinary behaviour (4, 5). Statistical evidence on antibiotic resistance and sales in reduction data in the UK is annually produced by the Veterinary Medicine Directorate (VMD) in the UK Veterinary Antibiotic Resistance and Sales Surveillance (6) reports. The UK-VARSS reports have become important surveillance tools used by policymakers and livestock industry stakeholders to assess and evaluate responsible use activities across the livestock sectors. The numerical “facts” in these reports are used to order problems, settle uncertainties, and govern the social (7). The latest UK-VARSS report of 2018 states there has been a significant reduction in AB-use across and within livestock sectors in the UK. From 2014 onwards, the UK has achieved a 49% reduction in veterinary AB sales (sold by pharmaceutical companies to veterinary practices) see Figure 1.

Problematically, different metrics and frameworks across and within countries have been advanced to help govern AB-use and AMR, which results in a lack of analytical frameworks to systematically understand AB-use and AMR reduction opportunities and targets (8). At the same time, Leach and Dry (9) reject the objectivity of scientific data models and observe a close interplay between science and politics that influence the processes of modelling. The politicisation of a problem, why it matters and to whom and what should be done about it co-constructs the development and preservation of these models (9). Equally, Barry (10, p. 270) argues how the authority of numerical evidence suppresses “potential places for contestation,” enabling debates to settle. Models and calculation become more than information; they serve as “anti-political” instruments to steer debates and settle concerns [(11), p. 208]. Moreover, some elements of an issue at stake are difficult to calculate and escape the metrological gaze: “calculative realities” are “thin” descriptions of reality [(12), p. 58]. Standardised procedures of models are not able to represent the complexity of the object and its practices in action [(10), p. 275]. As a result, AB surveillance models only partially represent reality as some aspects of AB behaviour are difficult to calculate and standardise and standardised procedures rarely accommodate the complexity of practices in action (10). This then ignores wider questions, such as how farmers and veterinarians are actually changing day-to-day antibiotic practices in response to policies.

In this article, we argue that there is a gap in the understanding of the design and workings of the UK's industry-led policy approach in practice. Taking the dairy industry as our main focus, we turn our gaze away from statistical evidence in favour of examining how responsible AB-use is executed from within the inner workings of the UK dairy industry. Using conceptual insights from Actor-Network Theory (ANT) (13) and the Sociology of Markets (14, 15), the aim is to understand how dairy supply chain actors make decisions about livestock AB-governance as a matter of concern according to *their* agricultural networks and how this translates into *doing* responsible use in a market environment. We argue that the UK dairy supply chain actor networks do not act in a vacuum when responding to their AB-use responsibilities as food supply chains; there are economic opportunities attached to the evidencing of responsible AB-use activities. This means there are limitations if we only assess antibiotic policies from *within* the existing UK policy framework. Although dairy supply chain actors produce the data required by the UK's industry-led policy frame, such as AB sales measured against reduction targets, this article will discuss how they tailor dairy AB policies and practices as well to interests *outside* these very same policy frames, with unintended consequences.

In what follows, we begin by explaining how antibiotic use in food animals is governed in the UK and introduce dairy supply responsibilities in the wider context of dairy supply-led milk safety and milk quality procedures. Next, we introduce ANT and the sociology of markets and explain our methodological approach to set the scene for our empirical data. Using fieldwork data, we discuss how UK dairy supply chains translate their responsibilities in policies and practices of these policies. We trace what is at stake in dairy supply chain AB policies and how this shapes dairy supply chain-led antibiotic policies. The paper ends with a discussion in which we express the need to move beyond behavioural regulatory frameworks and focus instead on structural needs of the UK dairy industry.

## Materials and Methods

### Setting the Scene: The UK's Industry-Led Policy Approach on Paper

**Figure 2:**
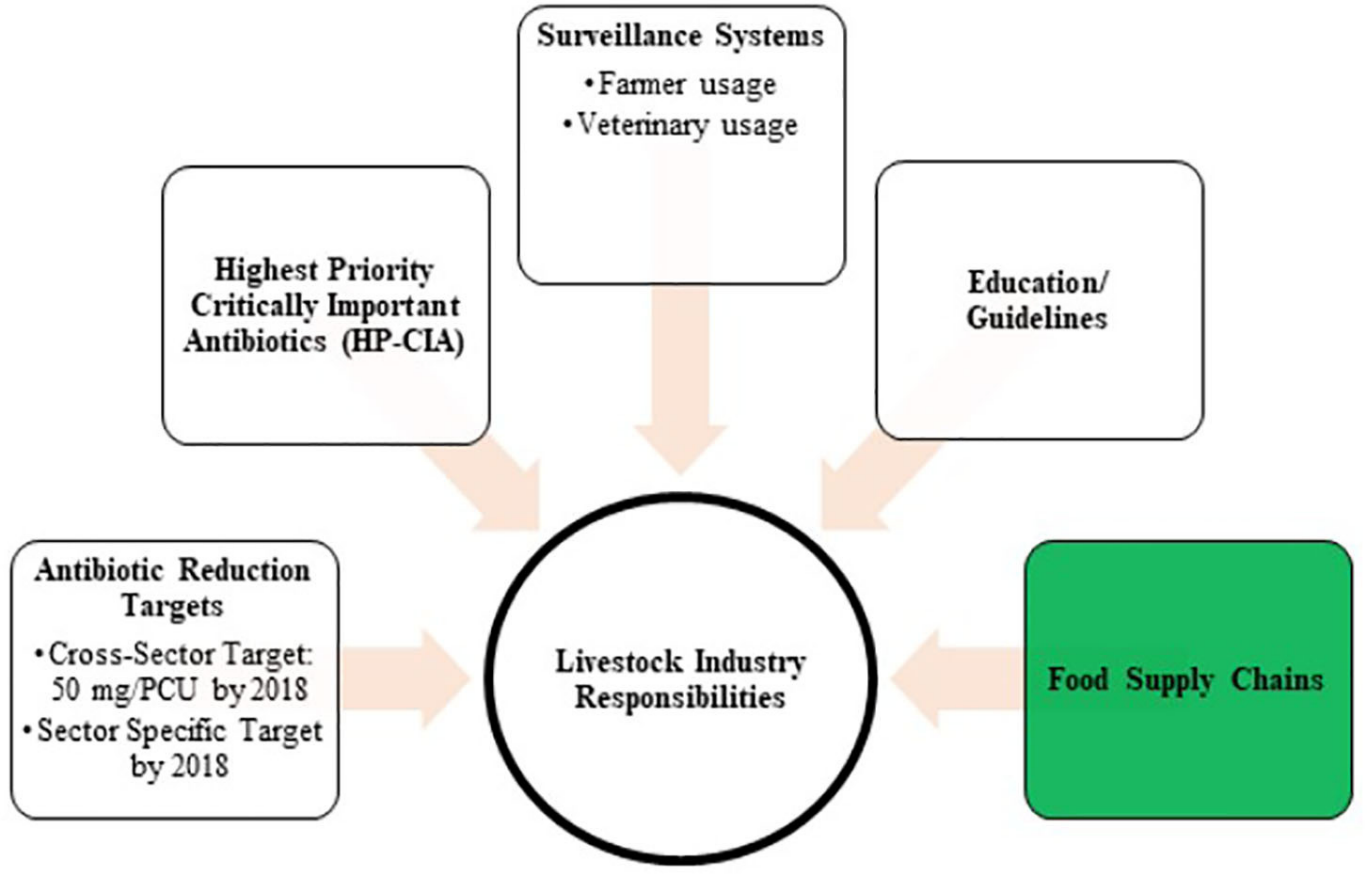
The UK's Industry-led approach to govern responsible use across livestock sectors (Department of Health 2016).

The problematisation of antimicrobial use in food animals in the UK started in the late 1960's and it has remained an issue of concern ever since (16, 17). What has changed in the latest governance negotiations in the UK between policymakers, experts and industry makers is that livestock sectors and their food supply chains have been made responsible for governing the issue (3, 4, 18, 19). The role of the UK Government is to monitor these responsible use activities through a national antibiotic sales surveillance system. The UK Veterinary Medicine Directorate (VMD) publishes the annual UK-VARSS reports to fulfil reporting requirements of the European Union / European Medicine Agency (20). These figures are also used to update the UK policymakers on antibiotic resistance and sales. The key targets of the UK's industry-led governance approach are presented in Figure 2 (4). In the context of the UK dairy industry, EU directives require that farms and processing plants undertake general hygiene measures related to the microbiological, physical, and chemical hazards of raw milk (21, 22). Through additional standards in terms of animal welfare, disease status, husbandry, and environmental footprints, private farm assurance schemes, milk processors, and retailers can add quality value to dairy products in highly competitive national and international milk markets (23). We will explain next how in the UK dairy supply chains, milk medicine residue management actors, the Dairy Red Tractor Farm Assurance Scheme, and milk processor and retailer milk contracts play a central role in definitions, expectations and practices of milk safety, quality and with it, antimicrobial practices.

One requirement in the EU directives is that animal products in the food supply chains are safe from medicine residues. When medicines are used in food animals, they can leave residues in animal food products (e.g., meat, milk, eggs) that could pose potential harm to consumers. To protect human health against AB residues in animal food products, AB withdrawal periods have been established for each AB product. The AB withdrawal period is the statutory minimum period that should elapse between the last day of AB treatment and the point at which the food-producing animal or its products enter into the food supply chain (24). Inside this statutory withdrawal period, food-producing animals, or their products cannot be used for human consumption. To limit residues in food animal products, supranational regulatory authorities have established “Maximum Residue Limits” (MRLs). The MRL is an official EU standard and is designed to protect the health of the European consumer by ensuring that food animal products are not placed in markets if they contain residues that their MRL (24). MRL thresholds of ABs are meant to separate AB “safe” from AB “unsafe” animal products. Although the VMD has a residue control program in place (25), the main responsibility for antibiotic residue management in dairy lies with the *milk processors* (and is, as such, industry-led).

To unify transparency across farm assurance schemes, in 2000 the British food industry introduced the “British Farm Standard,” known as Red Tractor. Established as a not for profit company. Red Tractor is owned, funded and run by the food industry and has become Britain's biggest voluntary private farm assurance scheme across livestock species. This form of third party certification, alongside official regulation, has been established at the European level as a mechanism to guarantee food safety from “farm to fork” (23, 26). The Red Tractor standards are species- or product-specific and stand for production standards covering the harmonisation of animal welfare, food safety, traceability, and environmental protection across food producers (27). During fieldwork in 2017; Dairy Red Tractor's AB quality standards were under revision, resulting in several new antibiotic standards (28–30). Importantly, establishment of the scheme creates the impression that ABs in dairy farms are being used in a responsible manner (23).

On top of the Dairy Red Tractor Scheme, UK milk processors and retailers use milk contracts with farmers as assurance systems which define the safety and/or quality conditions of milk production. Milk processors set standards in the milk contracts in relation to milk quality/composition (somatic cell count, milk solids such as fat-protein-lactose and mastitis bacterial pathogens such as *E. coli*, staphylococcus spp.) and milk safety (residue control). Farmers receive a milk price in accordance with the quality/composition of the milk produced, as specified in their milk contract. The price a farmer receives for milk depends upon the nature of the contract. In addition to these milk contracts with processors, some British retailers (Tesco, Waitrose, Sainsbury, M&S) contract directly with farmers. This usually means that farmers need to meet a set of retailer standards (including antibiotic standards) as well as those of the milk processors. The farmer will still receive a fixed milk price from the retailer. These “dedicated supply chains” allow retailers to increase their profit by selling milk under their own-label tailored to meet the needs of their consumers (31). Retailers may require higher standards of animal welfare, disease screening, husbandry and environmental footprints, which enable value to be added to their dairy products in a highly competitive milk market.

As will become clear in the results section, economic interests attached to milk residue management, red tractor standards and milk contracts shape the content, implementation and practices of antibiotic policies in the UK dairy supply chains.

### Theory and Methods: Theoretical Framework: Actor-Network Theory and the Social Studies of Markets

The UK food supply chains have been made largely responsible for the governance of responsible AB-use, in which metrics and knowledge transfer policies are used to govern and evaluate AB policies. As we argued, there are limitations attached in trusting scientific methods and their evidence to govern responsible AB-use in agriculture. Rather than accepting established risk frames, regulatory responsibilities, and granting scientific methods analytical privilege, we chose to examine multiple knowledges, conflicts, and interests attached to antibiotics in the UK's dairy supply chain's and how this co-constructed the practices of “responsible use” in the UK dairy sector. The latter is important, as it means that dairy antibiotic governance by dairy actors cannot be approached as an individual, rational act., but as a *collective* performance by networks of human (retailers, milk processors, farmers, etc.) and non-human actors (milk residues, Red Tractor Farm assurance Standards, Milk contracts, etc.).

The relations we build with human and non-human actors, which Latour (13, 32) refers to as actor networks in his Actor Network Theory (ANT), shape how we produce knowledge, and how this knowledge in turn, shapes the configuration of the actor-networks (33). Conceptually, ANT first of all bridges the nature/culture divide, by bringing non-human actors in their analytical frameworks, such as animals, technologies, literature, microbes, chemicals, institutions, laws, markets, and many more [(34–36)]. Secondly, taking scientific objects or technologies as objects of study rather than human behaviour, ANT scholars study the formulation of knowledge practices within and across sites of interests, in which the object circulates (in this case antibiotics) (13, 35). How knowledge is produced upon ABs depends what concerns (in terms of other actors) are attached to the ABs. For a farmer, ABs represents healthy cows and economic security, for vets ABs deliver farmer animal health/welfare and financial income, for retailers and milk processors, ABs pose a risk as milk residues, to consumer trust and unstable markets. As will be explained, different *matters of concern* circulate about ABs, depending upon the agricultural network they belong to.

Building on ANT, Çalişkan and Callon (14, 15) have studied markets as a socio-technical arrangement between human and non-human actors. Technologies exert an active role in this conceptualisation of markets. Through a process of “economization” (14), interests around technologies translate in new social, material, technical, and institutional arrangements between human and non-human actors as the market around the technology emerges. The work of Buller and Roe (37) on the process of economization around the issue of animal welfare in free range layer chickens is of particular interest here. The authors show that a rise in consumer agency has pushed retailers to incorporate consumer demands into their food market strategies. Evidence of “doing animal welfare” from “farm to fork” by food chain actors is represented by a range of “procedures, technologies, and performances” that “add” welfare standards to the chicken body [(37), p. 142]. In this way, the food chains' interpretation of consumer concerns shapes new markets and increases the power of the food supply chain over the bodies of animals (37). Using these ideas, we wanted to understand how dairy supply chains as economic actors conceptualise responsible use and how this results in new antimicrobial standards, protocols, descriptions, and technical devices.

Importantly, market actions inevitably contain a process of “framing,” with not only intended but also unintended effects or “overflows” of actions on other agents (38–40). The concept of framing/overflowing is important in our analysis, as it will not only help us to examine what “matters of concern” drive policy action, but also how this results in unintended effects or “overflows.” As Callon et al. [(39), p. 236] argue, “markets, when calculating interest, profits, and return on investments, draw a strict dividing line between that which is taken into account and that which is not.” To study the “matters of concern” and how this is translated in the dairy supply chain governance of responsible use, emerging markets and its overflows, we asked the following questions: What actors make up the matters-of-concern involved with responsible use across dairy supply chain actor? What new dairy markets emerge as a result of new socio-technical arrangements around responsible use governance in dairy supply chains? What are the unintended effects or “overflows” of the UK dairy supply chain governance of AMR?

## Methodology

ANT researchers use an ethnographic approach to study knowledge production and practices across sites of interest (35, 36, 41). Traditional ethnographies use observational methods to study people's actions and accounts in everyday contexts instead of experimental settings (42). They are most often single-sited ethnographies, demarcated in advance to study the situated human experience for a certain period of time in a setting of interest. Doing ethnography in ANT involves a different methodological approach than classical ethnographies. Their research tends to be multi-sited, examining the variety of knowledges across sites and actors instead of being limited to scientific experts in their laboratory settings (42). As our ethnographic interests were situated in AB knowledge practices (35), the handling (politically, economically, or physically) of livestock/dairy ABs could be expected to differ between sites. We traced livestock/dairy ABs to the sites where *they* brought us, including both human and non-human actors, ranging from retailer-farmer meetings, to farms, to people, livestock organizations, policy documents and more. The research did not involve finding truth, but instead describe how truth upon ABs is “enacted” or “performed” (35, p. 33).

Ethnographers often employ a variety of qualitative methods, tailored to the demands of the research site(s). This strategy is sometimes referred to as triangulation, in which more than one method of data collection is used (43). The methods usually involve observation (participant or non-participant) as the main method (covert or open), complemented with other methods such as in-depth interviews, focus groups, the study of material (documents) and may include quantitative approaches such as questionnaires (44). In this study, triangulation was used in a flexible manner in accordance with the sites of a multi-sited ethnography. This meant that in some sites we requested documents, interviews, participant-observations in veterinary clinics and on farms, focus groups or a combination of any of these. In order to get access to dairy stakeholders, we initially used social network of the University of Liverpool to recruit dairy supply chain actors, farmers, and veterinarians. We than opted for a snowballing or referral technique, where we recruited future participants from the social networks of the participants we interviewed. A recruitment advertisement was moreover published in a popular veterinary magazine to invite veterinarians to participate, but this resulted in only one positive response. We also encountered difficulties to get access to the big dairy companies, which resulted in a small sample of milk processor and retailer representatives (for a full oversight see Appendix 2: list of ethnographic fieldwork).

During the period of the ethnographic fieldwork between January 2017 and December 2018, 10 retailer-farmer meetings across the UK were observed. Next, 4 weeks of participant observation were undertaken at two veterinary clinics. This involved work shadowing of vets during their daily activities in the veterinary clinic and accompanying them during farm visits. A research diary was used to make notes of observations and discussions with veterinarians and farmers. Semi-structured interviews were furthermore undertaken throughout the course of the fieldwork with 4 key agricultural/dairy organisations;4 dairy supply chain actors; 2 veterinary consultants working for dairy supply chains as well as 21 veterinarians not directly connected with the supply chain and 3 farmers. Two focus groups were also conducted with retailers and farmers (see Appendix 2 list of ethnographic fieldwork). Interviews focused on dairy supply chain's understanding of responsible use, AB concerns, the policy process, and responsible AB-use practices (see Appendix 2: interview guide). The interview material and transcribing records were kept on a secured hard drive space only accessible through passwords.

Data analysis evolved around the generation of data from the fieldwork material instead of using pre-determined models. This inductive approach allowed us to generate patterns, codes, and themes by the language and/or topics discussed by the respondents or from the observational data. The first step of data analysis was transcription of data in textual record (45). In order to situate actor-networks that were emerging throughout the fieldwork, we transcribed interviews as close to the time they were undertaken as possible. For the data analysis, we used N-Vivo as coding tool and performed a thematic analysis, which involved the identification of recurrent themes in the data that organized our topics of interest. Data was “reduced” in labels or codes which helped to start comparing transcripts and enabled us to bring in STS theories and concepts that supported the data. In what follows, we will represent the results of data analysis.

### Results: The Practices of AMR Governance in the UK Dairy Supply Chains

#### Milk Processor Concerns: Milk Residue Governance

We identified that an important element of milk processors' responsibility to deliver milk safety is through medicine residue management. From a food safety perspective, the EU MRL legislation requires that products testing MRL positive at *individual cow level* should be recalled and destroyed (46). Importantly, it is the farmer who is responsible for ensuring that the raw milk of the *individual* cow is safe before it enters into the bulk milk tank (46). Milk processors rely on these responsible AB-use practices by farmers to reduce the risk of AB residues entering the milk supply chain. However, when the milk in the tanker travels from farm to farm before reaching the milk processor plant, it becomes diluted with milk from other cows on other farms. Similarly, milk residues get diluted when travelling from milk tanker to large milk tanks at processor plants. Milk processors refer to this process as “natural dilution,” which occurs as a result of operational procedures (Interview milk processor 2). In this way, raw milk that exceeded MRL levels at individual cow level can test below MRLs at processor level because of this “natural dilution” process. Regardless of whether the milk can be considered safe, technically the farmers and milk processors are officially in breach of the EU MRL law if the MRL levels are exceeded at individual cow or farm bulk milk tank level.

“*So natural dilution, it is not deliberate dilution but it is an operational consequence. We can't dilute out residues deliberately, it will be illegal to do that”* (Interview milk processor 2).

To protect dairy supply chains, the FSA has previously tolerated the “dilution-effect” of milk residues by turning “a blind eye to it” (Interview veterinary surgeon 21). The recent international politicisation of the public health risks of AB-use in food animals has, however, renewed attention on how the UK dairy industry manages AB residues (Interview milk processor 2). According to some dairy industry stakeholders, the FSA has now begun to exert pressure on milk processors to increase their milk residue controls (Interview veterinary surgeon 21, respondent livestock organisation 4). This has turned milk residues into a matter of concern for some of the UK milk buyers, not only from a milk safety perspective, but also financially. Milk processors incur considerable expenditure to investigate milk failures (Interview milk processor 2). For milk processors, responsible AB-use by farmers can reduce the risk of AB residues entering the milk supply chain (Interview milk processor 1 and 2). Farmers are, in this case, identified as “key actors” who need to be educated so they can take up their individual responsibility for managing the risk of milk residues.

“*Our main drive is to ensure that we don't have any antibiotic contamination in our milk. So we work very hard with our farmers to ensure that if they use antibiotics, they actually make sure they test their own milk before that milk goes into the milk factory for collection by ourselves”* (Interview milk processor 1).

“*What we are seeking to do is reducing the number of occasion where testing reveals the presence of antibiotics, so we are trying to develop a strategy that will help farmers to improve their performance in that respect”* (Interview respondent livestock organisation 4).

Milk processors identify two important antibiotic practices by farmers that can result in milk residues entering in the dairy supply chain: “dry cow therapy” and farmers' “off label use” of antibiotics. Some milk processors (Interview milk processor 1) believe half of the antibiotic use on dairy farms occurs during the drying off period. The dry cow period is the part of that cow's lactation cycle during which the cow's milk production is stopped for at least 40 days until the next parturition (47). Previous guidelines recommended the blanket treatment of cows with ABs in the dry period, and it has since become the biggest source of prophylactic AB-use. An alternative strategy is Selective Dry Cow Therapy (SDCT), in which cows with a low probability of infection are given a teat sealant to prevent pathogens entering the cow's body (48). This strategy significantly lowers the use of antibiotics. As such, some of the UK milk processors see SDCT as the most important strategy to reduce AB usage of their farmers and with it, the risk of milk residue failures (Interview milk processor 1 and 2).

“*If you can influence the strategy that farmers use in drying off, that is the way you can have the biggest impact on antibiotic usage”* (Interview milk processor 1).

“*There has been a real push for selected dry cow therapy from the local dairies”* (Interview veterinary surgeon 5).

Farmers' off-label use of ABs is another major concern to milk processors. Going off label means using medicines outside the terms of the licence, which can affect the milk withdrawal period. One vet suggested that some farmers do not understand these off label mechanisms or misuse, which increases the risks of raw milk residues entering the milk supply chain (Interview veterinary surgeon 21). Farmers often prolong AB treatments or give an “extra shot” which prolongs official withdrawal times of original treatments (topping up effect).

“*They don't necessarily know what going off-label is, because they might have never actually read what the on- label treatment is. And they also don't always realise that by going off-label, they are increasing the risk of residues because of the topping-up effect”* (Interview veterinary surgeon 21).

From an ANT perspective, milk residues as a “matter of concern” to milk processors and its related practices such as dry cow therapy and farmers off-label use shape how milk processors respond to antibiotic governance. Milk processors not only produce political evidence of antibiotic governance, but also build new economic relations with retailers by addressing milk residues as economic risk to dairy supply chains. In their AB strategies, milk processors have made farmers accountable and foregrounded farmers individual responsibility to deliver safe milk. Policy instruments of behaviour change, such as SDCT protocols and workshops, milk residue stewardship programs, milk price penalties, and AB test kits are implemented to bring AB practises into line with established protocols. The antibiotic policies are supposed to reduce the risk of milk residues entering dairy supply chains. In practice, science and its methods are not transferred through a linear process. A clash can often be observed between the evidence-based theory of protocols/ guidelines/standards and the reality of the professional decision making processes [(49), p. 1083; (50)]. Following Hamilton (51, p. 4), who is equally critical of evidence-based approaches that aim to bridge the theory-practice divide in vet-farmer communities, we will discuss how knowledge is not a product requiring discovery, communication and uptake. The adoption of knowledge tools emerges from a negotiation with local cultures and environments (52). In the next section, we will show how knowledge gets tweaked and tinkered with in accordance with matters of concern in farmer's agricultural networks.

#### Farmers Practices of Milk Processor Antibiotic Policies

Farmers identified the differences between artificial workshop settings and farm realities. While artificial classroom settings fostered communication and knowledge exchange between farmers it did not reflect the realities of working on a farm. As one farmer argued “you need to get mud on the boots and get out there” when learning new farming practices. Moreover, farmers argued that the act of SDCT is a process, entangled with conditions on the farm (Interview farmer 1). The milk processor SDCT protocols/workshops fail to address these complexities as they reduce the act of SDCT into a technical performance. Equally, veterinarians argued that knowledge transfer tools such as protocols, training, and videos were not always adopted by farmers in the ways that industry policymakers anticipated. Pre-existing values and reluctant attitudes toward technocratic interventions resulted in resistance toward milk processor antibiotic policies.

“*The nature of farmers as well is you are dealing with people that have been doing things for a long time but don't always think what they are doing is wrong. So trying to take people, farmers, who see that they have a problem in the first place, seeing where that problem might be coming from, seeing what they could do about that problem and then actually doing something about it is a…you have farmers on many different stages in that process”* (Interview veterinary surgeon 5).

“*Sometimes people are just very, very busy. Like it is hard to change your protocol and policies […] that is why we have always done it cause dad did it. But it is hard to change that because it takes time and effort and thought and if you are already working at full capacity…it is really difficult to change that. And some of them just don't have the money”* (Interview veterinary surgeon 2).

An unintended effect of AB self-tests is that farmers are becoming more knowledgeable about antibiotics and use them strategically to avoid positive milk residue tests, as farmers get penalised in case of a positive case. Instead of implementing appropriate AB-use behaviours, the policy develops a culture of what farmers can get away with. They use the tests as a “fruit machine game,” to find the earliest data when the milk can be put back in the bulk milk tank (Interview veterinary surgeon 21). The penalising systems and control systems of milk processors produce reactive responses of farmers instead of systemic changes in their AB practice. But farmers should not be blamed for their policy responses, as they are constrained by the dynamics of their own agricultural networks. AB policies focusing on “behavioural” change rather than addressing structural problems in agricultural communities will therefore fail to change AB practices on farms. The use of on-farm milk residue tests has moreover resulted in farmers disposing of more waste milk into the environment than before (Interview milk processor 1). Until recently, this waste milk was usually fed to calves. However, under Red Tractor guidelines waste milk should no longer be fed to calves to avoid the risk of AB residues surfacing in another part of the food supply chain (28).

*The truth is, they are probably disposing more milk than they have ever before. If they have an accident, it is much more beneficial for them to just get rid of the milk, as opposed to be charged 10p if they are found to be in breach”*(Interview milk processor 2).

With waste milk ending up in agricultural lands or in slurries, this potentially creates a new uncontrollable pathway of milk residues. In this way, creating a system focussing solely on protecting the food supply chain from milk residues, “co-produces” a potential unintended environmental cost.

Finally, during our fieldwork various respondents (farmers, vets, consultants, and milk processors) were keen to emphasise some of the limitations of the Red Tractor farm assurance scheme. They raised questions about whether the Red Tractor Standards actually deliver what they promise on paper.

Although Red Tractor is meant to stimulate best farming practices, not all farmers saw the value of the assurance system. Respondents suggested that the Red Tractor scheme allows farmers to approach their Red Tractor obligations as a “tick box” exercise rather than as a tool that supports innovation.

“*That is one of the problems with farm assurance, you know, a lot is a tick box exercise […]. And if farmers do it by tick boxing they are probably not doing it for the right reasons. And we have people like that, of course we have that. It's hopefully at the time it will be less and less of them”* (Interview milk processor 2).

“*When you do your Red Tractor assessment they don't measure any barrier space or cubicle space. They don't count your cows or your cubicles. They have all recommendations of what should be but nothing is actually controlled. It is a paperwork exercise, tick a box, as long as you meet the major compliances. It is not good. Red Tractor is not good. It is the basic standard. Which a lot of farmers would say ‘well yeah, but don't burn yourself with cost’. That is a fair comment, because cost drives down profit”* (Interview farmer 2).

We found that resistance toward paperwork resulted in some of the farmers falsifying their records.

“*What really struck me was, this farm was filthy. The house was filthy, the bathrooms, everything was absolutely filthy, he was filthy. This record book was immaculate, absolutely immaculate, not a spillage, nothing on it. Same pen and that was really good. I said about this when I went back in. So he showed me the calving issues, it was on the computer and I looked at it and he had written it out in the wrong month. So he had obviously written it before we came, done all the right cows and everything else, all the treatments but done all the records in the wrong month. So he transcribed it wrong. So that was the end of that. It was falsified records”* (Interview milk processor 2).

For some farmers, the Red Tractor scheme and milk processor standards produce a “paper reality,” in which there is a difference between what farmers *record* on paper and what they *do* in practice. Escobar and Demeritt (53) have found similar issues around audit, assurance, and animal welfare regulation on livestock farming, in which the materiality of paperwork produced resistance of farmers, resulting in failure of compliance. As such, rather than constructing farmers as “naïve” adopters [(54), p. 1776], we need to understand how farmers respond to and integrate milk quality and milk safety standards of private assurance schemes into their local values, customs, and practices.

#### Retailer Concerns: Consumer Profiles

From 2010 onwards, extensive media attention and consumer concerns have pushed some of the major retailers to implement AB policies, with these policies mainly focusing on recording AB usage (Interview retailer 2, veterinary surgeon 2). The publication of the O'Neill report in 2015 and the media concerns that came with it refocused retailer's attention on the issue (55). Retailers feared media messages could threaten consumer trust in food supply chains. From 2016 onwards, to avoid negative publicity, most UK retailers have implemented antimicrobial policies across their food supply chain. The dairy industry is considered as a particular risk to retailers, as the industry lacks oversight on how dairy farmers operate (Interview retailer 2).

“*Retailers feel they have a far more vulnerable relationship with their dairy supply chain than they do with their beef and chicken supply chain for example […] There is a massive range of dairy farmers. You've got some really good operators, and you've got some shit operators. So that makes them feel vulnerable. So, retailers want to be able to show they are doing something, working with their farmer suppliers to reduce the routine use of antibiotics”* (Interview veterinary surgeon 21).

What matters to retailers is that they implement antibiotic standards that result in “evidence” of responsible antibiotic use activities. Metrics on AB reduction numbers are of particular interest to retailers, as this is visible evidence and provides accountability to consumers that responsible AB-use is being performed by farmers.

“*From a retail public facing point of view is that, the public is only interested whether you use antibiotics or you don't. I think in dairy that is a challenge, but what we are doing now is restricting it and having the evidence that we actually have restricted it you know, so you always have it in your toolbox”* (Interview retailer 1).

However, at the time of this fieldwork, retailers with customer *quality* sensitive profiles defined antibiotic standards and herd health standards that exceeded milk processor and Dairy Red Tractor Scheme standards. These retailers had their own teams of experts who translated consumer expectations into milk contract expectations. Additional policies and knowledge transfer tools on HP-CIA and SDCT, on top of milk processor and Dairy Red Tractor Scheme standards, were implemented at the time this research was performed. Some of these retailers used “interactive” sessions across the country to relay the new policy to farmers and vets. It was believed that these interactive meetings would foster farmers' adoption of the HP-CIA policy (retailer 2).

*What we have done in January is that we focused on the mind of farmers, we don't believe that we have moved far enough yet and therefore we should encourage farmers to make further steps to improve […] even though the farmers have been aware of it the last 6 years, there hasn't been a massive swing away from CIAs, that we could show in our results. So the feeling was we needed to communicate back and re-focus our efforts in trying to encourage farmers before there was legislation”* (Interview retailer 2).

Some retailers increasingly push farmers to record herd health and welfare activities on their farms. The herd health performativity of farms is used to identify areas of improvement. Retailers can use this information to promote good husbandry practices in their food supply chains to consumers. Importantly, herd health performativity has become increasingly linked with AB performance on farms by retailers.

“*I make sure that it is a benefit to farmers to record the information around health and welfare and recording the use of antibiotics, and other medicines, and recognize health as a responsibility, but also as a benefit to the farm, so they can benchmark themselves […], that information is stored within our database and number crunched, that gives us a score of each farm. We can than benchmark each farm one against another, and use that information to develop strategies for those farmers who we believe and we can identify using far more antibiotics than the average or good dairy farms”* (Interview retailer 2).

Herd health performativity standards moreover enable new commercial platforms to emerge for those retailers who are chasing differentiation in milk quality with their contracted farmers.

“*Retailers are given a competitive marketplace and they are all seeking to find whether there is an opportunity for commercial advantage, so retailers and processors have their own policies and strategies on this”* (Interview respondent livestock organisation 4).

“*They want their own schemes, they want their own control, they want their points of differentiation. and I think, to a certain extent, that will be true, er, you know, of antimicrobial usage policy”* (Interview veterinary surgeon 20).

Other retailers with customer *price* sensitive profiles may not have a “dedicated” milk pool. This means they are not directly involved with the production standards under which farmers produce. In this case, it is the milk processors and Dairy Red Tractor Scheme which sets the quality assurance of milk production and defines how dairy products take shape from farm to fork.

The previous fieldwork data shows how consumer concerns shape retailer responses on the governance of responsible AB-use. A process of “economization” takes place in which antibiotic standards potentially become part of commercial retailer strategies to position themselves on dairy markets. As a result, antibiotic policy strategies such as knowledge transfer strategies, SDCT and HP-CIA standards and herd health expectations differentiate rather than unite antibiotic practices by farmer and veterinarians. Rather than collectively addressing responsible use, retailers have their own expert teams, strategies, and priorities attached to responsible use.

Some researchers have questioned the neoliberal form of food safety governance by food supply chains. Higgins et al. [(54), p. 1778] have argued that this type of governance in food supply can “reinforce the profit-making logic of capital” instead of being translated in hegemonic practices. Bailey and Garforth (23) have also discussed how private industry led standards are used by food supply chains to gain marketing advantage and brand promotion. Nevertheless, from *within* the UK's industry-led framework and inside market boundaries, retailers are able to provide metrics and anecdotal evidence of success. However, we will show how the antibiotic policies represent only a thin reality of what happens *outside* the realm of the UK's policy framework and markets, with unintended effects.

#### Farmer Practices of Retailer Antibiotic Policies

During fieldwork and interviews, it became clear that the metrics around responsible use activities only provides a snapshot of reality. Some of the farmers contracted by retailers with customer quality sensitive profiles tried to circumvent contractual obligations.

“*The farmer says that farmers are not filling in the files correctly. Some farmers complete online retailer quality assurance forms as the best performers but in the meantime, they use different billing systems for their antibiotic registration or use their secret stocks”* (Fieldnotes veterinary practice 3).

“*I had this case a farmer said to me that he was going to Ireland to buy in a lot of marbocyl”* (Interview veterinarian 14, fieldnotes veterinary practice 3).

“*The farmer may give penicillin as it is the first dose and he may complete the course brilliant. But another farmer may think well I know that the cephalosporin works better, in his mind, and he will give the penicillin, and at the same time give the cephalosporin. So on paper, it looks like they have done it wright, but in reality…they have given 2 drugs at the same time for no apparent reason”* (Interview veterinarian 17).

At the same time, farmers saw attendance as an obligation associated with their milk contract. Farmers are evaluated on whether they attended retailer meetings and workshops and therefore make an effort to show up at meetings, but instead of engaging with the knowledge-transfer, some farmers saw the retailer farmer meetings as a nuisance that disrupts their day. Although retailers may believe on the basis of attendance that they have successfully transferred their policies through interactive sessions, farmers will still continue with their daily routine practices as is expressed in the following quote:

“*At the moment we seem to have series of workshops where we go to a nice hotel and we have a nice lunch and we have four speakers who talk to us about varying things. And we all fill a form in after, and we all go home and carry on and do what we were doing before. It was described to me the other day as: “Are you confident in what you're doing?” This was another farmer. He said, “Are you confident in what you're doing?” He laughed as he said it. I said, “Well, yes.” He said, “Well, do what I do.” I said, “What's that?” He said, “Let it wash over you, tick the box, move on”* (Interview farmer 2, recorded in fieldnotes at retailer farmer meeting 9).

Participatory policy making as strategy to develop AB policy has been recently studied by van Dijk et al. (56). The process involved collaboration and dialogue between producers, veterinarians, industry, and researchers. Farmers and veterinarians in this case were seen as active partners in collaborative decision-making (56). Although the retailer we work shadowed during retailer farmer meetings used participatory policy making as approach, some of the farmers and vets argued that they did not felt enough included in the policy process, resulting in reluctance toward the retailer policy approach.

“*Their policies involve a lot of tick boxing, but nobody actually reads the documents. We don't feel understood, included in the program. I feel it is us against them”* (Discussion with farmer, recorded in fieldnotes at veterinary practice 3).

“*I think as vets in practice we have not necessarily kept in the loop beforehand, so farmers have been going to retailer meetings and we have not necessarily found out about some of these things until after fact so we have not been able to take the initiatives as much as we've liked […] and it means that if farmers hear from the supermarkets in their contracts that ‘oh right we have to start doing selective dry cow therapy or at least have a look at it’ and they start doing that without adequate teat preparation and hygiene, well we had plenty of farmers that have lost cows as a result of that. Whereas if vets had been involved beforehand then perhaps we could have implemented some proper training”* (Interview veterinary surgeon 5).

The different retailer contractual expectations on responsible AB-use resulted moreover in different antibiotic practices of veterinarians and farmers.

“*We had a bad caesarion and normally we give marbocyl to bad operations, but now we give it synulox. However, this client was not contracted with retailer X and he wanted marbocyl. The thing is with marbocyl is that you see a very quick recovery and milking gets up quickly. Which is not the case often with other drugs. So in this case we put the cow on marbocyl, the calve lived and we had a good overall recovery of the animals and productivity. I think supermarket contracts do influence antibiotic choices as you need to justify your use. If you know the retailer contract and expectations, you adapt your choices”* (Interview veterinarian 14, recorded in fieldnotes veterinary practice 3).

“*They are still using Marbocyl, as they are not on a specific retailer milk contract that does not ask them to reduce use”* (Interview veterinarian 13, recorded in fieldnotes veterinary practice 3).

The previous discussion illustrates how the different contractual obligations/expectations of retailer milk contracts segregates dairy farmers and vets in their AB practices rather than unifying them. An important message from the previous discussion is that farmers and vets respond to dairy supply chain AB policies in accordance with *their* actor-networks, rather than it being an individual act. More research is therefore needed to examine how local agricultural networks shape farmers and veterinary antibiotic practices, rather expecting the individual farmer and veterinarian to change behaviour.

## Discussion and Conclusion

We have revealed how the formulation, implementation and adoption of responsible AB-use within the UK dairy industry is interactional and interpretative, located within and across dairy supply chain actor-networks and their concerns. Using the methodology of examining the dairy antibiotic policies and practices of the UK dairy sector, we demonstrated how different matters of concern regarding dairy ABs circulate, affecting antibiotic policies and their practices. By tracing ABs in their actor-networks, we found which dairy actors are of importance and how this steers AB decision-making: how milk residues drive milk processor policies; how customer profiles drive retailer policies; and how agricultural interests define Red Tractor farm assurance standards. To farmers, AB decision-making is situated in complex agricultural networks; milk prices, milk contracts, milk withdrawal times, and more. We argue that antibiotics have become integral to farmers' understandings and practices of animal health and animal performance. ABs are embedded in the daily practices of farmers, supported by a vast array of human and non-human actors that confirm their importance. Educational strategies, training programmes, and technologies that support antibiotic governance will have a limited impact in changing farmers' behaviour, as long as antibiotics remain accessible to farmers.

The understanding of actor-networks is crucial if we want to evaluate how UK dairy supply industries take up their responsibility in terms of AB governance. In fact, our findings emphasise that AB-use is an inherent dairy supply chain economic activity, instead of an individual *choice* of farmers. We showed how AB decision-making, in terms of policies and practices, is situated in market interests of the dairy supply chain actors. Moreover, through a process of economisation, antibiotic standards translate into dairy cows, metrics, farmer behaviours, and new market opportunities. However, with these emerging markets strictly defining what to take into account and what to ignore in terms of responsible AB-use (to maintain maximum output in their economic actor-networks), they generate exclusions and overflows (39). We have demonstrated in this article that dairy supply chain AB policies are not only practiced in accordance with the economic interests of milk processors and retailers, but also in accordance with farmer realities, potentially “co-producing” new potential invisible environmental, foodborne and human routes of AMR. Consequently, by making livestock industries responsible for implementing AB policies, without taking into account what “matters of concern” drive their AB decision-making, AB policies and their practices will create unexpected pathways and outcomes.

Having exposed the complexity of dairy AB actor-networks and their overflows, it becomes difficult to believe we can transfer antimicrobial policy responsibilities solely to the UK dairy industry. Neither can we expect farmers and veterinarians to ignore their AB interests if there are no financial alternatives offered. We suggest that the problem is not AB-use in itself; the “misuse and overuse” is merely a symptom of a dairy sector in need of structural changes at multiple levels. In order to re-evaluate our policy frame and interventions, we need to engage with the complexity of dairy AB networks rather than wanting to reduce it. This involves examining “matters of concerns” at multiple levels, from veterinarians, farmers, food supply chains, governments to consumers, and how they “co-produce” each other. Interventions need to simultaneously address these multiple levels to reduce the risk of overflows. Moreover, we need to continuously work *with* overflows of interventions and tackle them rather than ignoring them.

One way to approach the limitations of today's approaches is to change how we problematise issues related to responsible antibiotic use. The way we formulate problems around the actor-networks that involve responsible use, whom we include in the responsible use research collective and how we disseminate and implement the results, requires different forms of knowledge organisation. Although farmers and vets are asked to give their feedback on the policies during retailer and milk processors, farmers, and vets are not included during the formulation of problems. But problems are not the monopoly of experts [(39), p. 77]. As Callon et al. argue [(39), p. 35], “what is at stake for the actors is not just giving their opinion or expressing oneself or exchanging ideas, or even making compromises; it is not only reacting, but constructing.” For example, what structural changes do farmers need in order to become less dependent upon antibiotics? What are problems or phenomena on farms identified by farmers that inhibit innovation and change in antibiotic dependency? Rather than contrasting lay knowledge and expert knowledge by referring to terms like rationality and irrationality, objective knowledge and subjective beliefs [(39), p. 80], we need a collaboration between different types of antibiotic knowledges.

It also involves vision-building across sectors and disciplines to study AB-use as part of a bigger picture of animal welfare, environmental impact and sustainable food production. Moran (8) has for example proposed to design a common framework across clinical, agricultural, and environmental settings that prioritises AB interventions on the basis of their cost-competitiveness. It moreover requires an analysis that acknowledges and integrates the different dimensions, levels, and stakeholders' interests associated with the problem under review. At the same time, the role of the state in facilitating schemes or monitoring the industries self-regulation of responsible use should be an important question to consider. As Higgins et al. [(48), p. 1778] argue, “the intermingling of private and public forms of governing is perhaps inevitable in dealing with environmental externalities generated by competitive agriculture, where meeting the contradictory demands of the market and public pressures to tackle environmental problems poses intractable dilemmas.” A limitation of this paper is that it focused on specific networks and policies at the time of research. As these networks and policies are changing, there it the risk that the empirical data does not reflect the dairy industry anymore at this present moment or in the future. Another limitation is the small sampling size of several stakeholder groups. Due to restrictions in time, our sampling data of each group was small, which means the voices we used to represent different stakeholder groups may not represent the wider view of each group reported on.

One topic we left unexplored in this article are the potential effects of the UK leaving the European Union. Being part of Europe means being part of their internal food market with food products produced against certain minimal standards. Although food standards come with overflows, leaving Europe means leaving a framework that tries to supports good husbandry practice, to the consumer, the animal and the environment. Brexit means entering new competitive agricultural markets with potential disruptions to the internal market. How will animal health and welfare, consumer expectations, and export positions be translated in food safety and quality? How can the UK compete with markets which have significant lower animal health and welfare standards? How will it set import and export tariffs without disrupting national markets? How will it patrol its borders to avoid the risks of importing non-native livestock diseases? These are just a few questions which will need to be addressed if the UK leaves the EU.

To conclude, we find that policy and science offer a reductionist way of seeing the world. Dairy AB-use gets boiled down to an issue of “overuse and misuse,” which results in a self-fulfilling prophecy: if only we measure we can see how effective we are. Within this frame, overflows don't matter because the frame has been set only to examine the use/misuse in relation to veterinary and farmer practices. However, in order to be effective, we have to look at the whole dairy supply chain network. The question next becomes how we can study AB-use as part of a bigger picture of animal welfare, environmental impact, and sustainable food production. Further research projects should therefore address the complex economic relationships which underpin food production, explore environmental concerns, include public views, examine the overflows of responsible AB-use policies, compare country approaches, and more. But for now, with the uncertainty of Brexit and a UK dairy sector in need for support and security, it is important to work together across levels to drive changes in the UK dairy sector as a whole.

## Data Availability Statement

The raw data supporting the conclusions of this article will be made available by the authors, without undue reservation.

## Ethics Statement

The studies involving human participant were reviewed and approved by the Veterinary Research Ethics Committee, Institute of Veterinary Science, Leahurst Campus, Neston, South Wirral, CH64 7TE. The patients/participants provided their written informed consent to participate in this study.

## Author Contributions

SB: writing the article. FW, IV, RC, and EP revisioning content. RV: editorial input.

## Conflict of Interest

The authors declare that the research was conducted in the absence of any commercial or financial relationships that could be construed as a potential conflict of interest. The handling Editor declared a past co-authorship with one of the authors RC.

## References

[1] WHO, FAO, OIE. Antimicrobial Resistance A Manual For Developing National Action Plans. Vol. Version 1. (2016). Available online at: https://www.who.int/antimicrobial-resistance/national-action-plans/en/ (accessed May 3, 2020).

[2] O'Neill J Antimicrobials in Agriculture and the Environment: Reducing Unnecessary Use and Waste. The Review on Antimicrobial Resistance (2015).

[3] O'Neill J Tackling Drug-Resistant Infections Globally : Final Report and Recommendations. London: The Review on Antimicrobial Resistance (2016).

[4] Department of Health Government Response to the Review on Antimicrobial Resistance September 2016. Available online at: https://assets.publishing.service.gov.uk/government/uploads/system/uploads/attachment_data/file/553471/Gov_response_AMR_Review.pdf (2016) (accessed May 3, 2020).

[5] RUMA Targets Task Force Report 2017. (2017). Available online at: https://www.ruma.org.uk/wp-content/uploads/2017/10/RUMA-Targets-Task-Force-Report-2017-FINAL.pdf (accessed May 3, 2020).

[6] UK-VARSS UK Veterinary Antbiotic Resistance and Sales Surveillance Report (UK-VARSS 2018). Addlestone: New Haw (2019).

[7] Mansnerus E. Using model-based evidence in the governance of pandemics. Sociol Health Illn. (2013) 35:280–91. 23278437 10.1111/j.1467-9566.2012.01540.x

[8] Moran D. A framework for improved one health governance and policy making for antimicrobial use. BMJ J. (2019) 4:807. 10.1136/bmjgh-2019-001807 31637031 PMC6768363

[9] Leach M Dry S. Epidemics Science, Governance and Social Justice. Routledge (2010).

[10] Barry A The anti-political economy. Economy Soc. (2002) 31:268–84. 10.1080/03085140220123162

[11] Maeseele P Hendrickx K Pavone V Van Hoywegen I. Bio-objects'political capacity: a research agenda. Corat Med J. (2013) 54:206–11. 10.3325/cmj.2013.54.206 23630150 PMC3641879

[12] Jasanoff S The ethics of invention: technology and the human future. New York, NY: W.W. Norton and Company (2016).

[13] Latour B Reassembling the Social. An introduction to actor-network theory. New York, NY: Oxford University Press Inc (2005).

[14] Çalişkan K Callon M Economization, part 1: shifting attention from the economy towards processes of economization. Economy Soc. (2009) 38:369–98. 10.1080/03085140903020580

[15] Çalişkan K Callon M The study of markets economization, Part 2: a research programme for the study of markets. Economy Soc. (2010) 39:1–32. 10.1080/03085140903424519

[16] Begemann S Antibiotic Policies in the UK Dairy Industry: A Voluntary Industry-Led Approach in Action. Liverpool: University of Liverpool (2019).

[17] Kirchhelle C. Swann song : antibiotic regulation in british livestock production (1953–2006). Bull History Med. (2018) 92:1–51. 10.1353/bhm.2018.0029 29961717

[18] RUMA Government Response to O'Neill Findings Welcomed amid Calls for Joined-up Leadership and Capital Investment. (2016). Available online at: http://www.ruma.org.uk/government-response-oneill-findings-welcomed-amid-calls-joined-leadership-capital-investment/ (accessed May 3, 2020).

[19] Alliance to Save our Antibiotics Alliance to Save Our Antibiotics Welcomes O'Neill Report and Calls on Defra to Set Ambitious Targets to Reduce Farm Antibiotic Use. (2016). Available online at: https://www.ciwf.org.uk/media/press-releases/2016/05/alliance-to-save-our-antibiotics-welcomes-oneill-report (accessed May 3, 2020).

[20] European Medicines Agency, European Surveillance of Veterinary Antimicrobial Consumption Sales of veterinary antimicrobial agents in 31 European countries in 2017 (2019). (EMA/294674/2019).

[21] Regulation (EC) No 853/2004 of the European Parliament and of the Council of 29 April 2004 Laying down Specific Hygiene Rules for Food of Animal Origine.

[22] Regulation (EC) No 854/2004 of the European Parliament and of the Council of 29 April 2004 Laying down Specific Rules for the Organisation of Official Controls on Products of Animal Origin Intended for Human Consumption (2004).

[23] Bailey AP Garforth C An industry viewpoint on the role of farm assurance in delivering food safety to the consumer: the case of the dairy sector of england and wales. Food Policy. (2014) 45:14–24. 10.1016/j.foodpol.2013.12.006

[24] FSA Milk Hygiene on the Dairy Farm A Practical Guide for Milk Producers. (2016). Available online at: https://www.food.gov.uk/sites/default/files/media/document/milk-hygiene-guide-for-milk-producers.pdf (accessed May 3, 2020).

[25] VMD Guidance Residues Surveillances. (2016). Available online at: https://www.gov.uk/guidance/residues-surveillance.

[26] European Commission White Paper on Food Safety. Vol. 1 Brussels: European Commission (2000). Available online at: https://eur-lex.europa.eu/legal-content/EN/TXT/PDF/?uri=CELEX:51999DC0719andfrom=EN (accessed May 3, 2020).

[27] Red Tractor Redtractor who we are/about-us (2020). Available online at: https://www.redtractor.org.uk/who-we-are/about-us/ (accessed May 3, 2020).

[28] Red Tractor Dairy Standards 1st October 2017 (Updated 1st June 2018) Version 4.1. (2017). Available online at: https://assurance.redtractor.org.uk/contentfiles/Farmers-6802.pdf?_=636790162726941131 (accessed May 3, 2020).

[29] RUMA New Red Tractor Antibiotic Rules Outlined (2017). Available online at: https://www.ruma.org.uk/new-red-tractor-antibiotic-rules-outlined/ (accessed May 3, 2020).

[30] Red Tractor Assurance Responsible Use of Antibiotics on Red Tractor Dairy Farms. (2018). Available online at: https://assurance.redtractor.org.uk/contentfiles/Farmers-6912.pdf?_=636585117784901746 (accessed May 3, 2020).

[31] Mylan J Geels FW Gee S McMeekin A Foster C Eco-Innovation and retailers in milk, beef and bread chains: enriching environmental supply chain management with insights from innovation studies. J Clean Produc. (2015) 107:20–30. 10.1016/j.jclepro.2014.09.065

[32] Latour B Science in Action. Cambridge, MA: Harvard University Press (1987).

[33] Sismondo S An Introduction to Science and Technology Studies. Wiley-Blackwell (2010).

[34] Murdoch J Inhuman/nonhuman/human: actor-network theory and the prospects for a nondualistic and symmetrical perspective on nature and society. Environ Plann D. (1997) 15:731–56. 10.1068/d150731

[35] Mol A The Body Multiple: Ontology in Medical Practice. Duke University Press (2002).

[36] Latour B Woolgar S Laboratory Life: The Construction of Scientific Facts. Princeton, NJ: Princeton (1987). 10.1515/9781400820412

[37] Buller H Roe E Modifying and commodifying farm animal welfare: the economisation of layer chickens. J Rural Stud. (2014) 33:141–49. 10.1016/j.jrurstud.2013.01.005

[38] Callon M An essay on framing and overflowing: economic externalities revisited by sociology. Sociol Rev. (1998) 46:244–69. 10.1111/j.1467-954X.1998.tb03477.x

[39] Callon M Lascoumes P Barthes Y Acting in an Uncertain World: An Essay on Technical Democracy. Cambridge, MA: MIT Press (2009).

[40] Barry A Slater D Technology, politics and the market: an interview with michel callon. Economy Soc. (2002) 31:285–306. 10.1080/03085140220123171

[41] Moser I Making alzheimer's disease matter enacting, interfering and doing politics of nature. Geoforum. (2008) 39:98–110. 10.1016/j.geoforum.2006.12.007

[42] Hammersley M Atkinson P Ethnography: Principles in Practice. 3th ed. London and New York: Routledge (2007). 10.4324/9780203944769

[43] Barbour RS. Checklists for improving rigour in qualitative research: a case of the tail wagging the dog? BMJ. (2001) 322:1115–7. 10.1136/bmj.322.7294.1115 11337448 PMC1120242

[44] Green J Thorogood N Qualitative Methods for Health Research. 1st ed. SAGE Publications Ltd (2004).

[45] Savin-Baden M Howell Major C Qualitative research the essential guide to theory and practice. Routledge (2013).

[46] FSA Information and Guidance on the Testing of Milk for Antibiotic Residues” 1 (2015): 1–25. Available online at: https://www.food.gov.uk/sites/default/files/media/document/testmilkantibioticres.pdf (accessed May 3, 2020).

[47] AHDB Dairy AHDB Mastitis Control Plan. Dry Cow Management: A Practical Guide to Effective Mastitis Control. (2020). Available online at: https://dairy.ahdb.org.uk/dry-cow-management/#.Xq0cSHduJhE (accessed May 3, 2020).

[48] Higgins HM Golding SE Mouncey J Nanjiani I Cook AJC. Understanding veterinarians' prescribing decisions on antibiotic dry cow therapy. J Dairy Sci. (2017) 100:2909–16. 10.3168/jds.2016-11923 28131572

[49] Berg M. Problems and promises of the protocol. Soc Sci Med. (1997) 44:1081–88. 10.1016/S0277-9536(96)00235-3 9131732

[50] Enticott G The local universality of veterinary expertise and the geography of animal disease. Transact Inst Br Geogr. (2012) 37:75–88. 10.1111/j.1475-5661.2011.00452.x

[51] Hamilton L Bridging the divide between theory and practice: taking a co-productive approach to vet-farmer relationships. Food Ethics. (2017) 1:221–33. 10.1007/s41055-017-0011-7

[52] Maye D Enticott G Naylor R Ilbery B Kirwan J Animal disease and narratives of nature: farmers' reactions to the neoliberal governance of bovine tuberculosis. J Rural Stud. (2014) 36:401–10. 10.1016/j.jrurstud.2014.07.001

[53] Escobar MP Demeritt D Paperwork and the decoupling of audit and animal welfare: the challenges of materiality for better regulation. Politics Space. (2017) 35:169–90. 10.1177/0263774X16646771

[54] Higgins V Dibden J Cocklin C Neoliberalism and natural resource management: agri-environmental standards and the governing of farming practices. Geoforum. (2008) 39:1776–85. 10.1016/j.geoforum.2008.05.004

[55] Morris C Helliwell R Raman S Framing the agricultural use of antibiotics and antimicrobial resistance in UK national newspapers and the farming press. J Rural Stud. (2016) 45:43–53. 10.1016/j.jrurstud.2016.03.003

[56] van Dijk L Hayton A VMD Guidance Residues Surveillances. (2016). Available online at: https://www.gov.uk/guidance/residues-surveillance.

