# Supplementary material for: The Governance of UK Dairy Antibiotic Use: Industry-Led Policy in Action
Source: Front Vet Sci. 2020 Sep 4;7:557. doi: 10.3389/fvets.2020.00557 (PMC7500462; doi:10.3389/fvets.2020.00557)
Supplement: Supplementary file 1 [file Table_1.DOCX]

**Interview Guide Industry England**

Thank you for taking the time to speak to me today. This conversation should take about an hour, but may take more or less time depending on how much you want to say. During our conversation, I may take a few notes but I will be recording the session on a digital voice recorder so I don’t miss anything important. I want to reassure you all your responses will be kept confidential. This means that any information you provide will only be shared within the research team, and we will ensure information is anonymised so you cannot be identified. You don’t have to talk about anything you don’t want to, and if you feel uncomfortable at any point and wish to take a break or end the interview, please let me know. If you have any questions before we start, please let me know.

Introduction:

| This interview is being conducted to get your input about the governance of antibiotic use in dairy farm animals and antimicrobial resistance (AMR) in England. I am especially interested in what your thoughts and concerns are on the topic, and how you think the uncertainties on the topic should be managed. |
| --- |
| **Interview Guide** |
| The opening section of the interview is intended to set the scene, initiate conversation with the participant, encourage them to feel at ease within the context and establish a rapport. |
| *Background and position of the interviewee:*   - Can you briefly describe your background? - Can you briefly describe your responsibilities in your current position? - How are you involved with antibiotics, animals, food and/or human health? - How do you think your background influences your thoughts and opinion on the topic?   *Policy:*   - What do you think of the UK Antimicrobial Resistance 2013-2018 strategy programme? - How is the government taking up its responsibility? - What do you think of other successful European policy programs (such as the Nordic countries, Denmark, the Netherlands). - How are the industry-sectors taking up their responsibility? - Are current policies on governmental and industry level making enough progress? - What is in your opinion a One Health approach?   *Antibiotics*   - What are your concerns on using antibiotics in food animals and the development of AMR in human health? - How should we use critical antibiotics in food animals? - What is the relationship between animal welfare and antibiotics? - What are your concerns regarding antibiotics and imported meat? - What is your opinion on changing husbandry systems and its impact on antibiotic use? - Do you take any precautions yourself towards the consumption of meat?   *Influence of other groups on the issue?*   - What do you think of the pressure from the media and the public? - What is the role of pharmaceutical industries? - What is the responsibility of retailers and supermarkets? - How should science inform the debate? |
