# Supplementary material for: The Governance of UK Dairy Antibiotic Use: Industry-Led Policy in Action
Source: Front Vet Sci. 2020 Sep 4;7:557. doi: 10.3389/fvets.2020.00557 (PMC7500462; doi:10.3389/fvets.2020.00557)
Supplement: Supplementary file 2 [file Table_2.DOCX]

**List of people interviewed (not in chronological order).**

| Interview list | Respondent |
| --- | --- |
| Livestock industry organisations | - **Livestock industry organisation 1 (Male, 50-65)** - **Livestock industry organisation 2 (Male, 40-55)** - **Livestock industry organisation 3 (Male, 50-65)** - **Livestock industry organisation 4 (Male, 50-65)** |
| Dairy processors | - **Dairy processor 1 (Male, 35-50** - **Dairy processor 2 (Male, 35-50)** |
| Retailer | - **Retailer 1 (Male, 50-65)** - **Retailer 2 (Male, 35-50)** |
| Pharmaceutical companies | - **Pharmaceutical company 1 (Male, 50-65)** - **Pharmaceutical company 2 (Male 50-65)** |
| Veterinary surgeons | **Veterinary practice 1 North West/Wales**   - **Veterinary surgeon 1 /researcher university of Liverpool, North West/Wales (Male, age 35-50)** - **Veterinary surgeon 2 North West/ Wales (Female 35-50)** - **Veterinary surgeon 3 North West/Wales (Female Parnter, 35-50)** - **Veterinary surgeon 4 North West/Wales (Male, Partner, 50-65)**   **Veterinary practice 2 North West**   - **Veterinary surgeon 5 North West (Male, 35-50)**   **Veterinary practice: 3 North West**   - **Veterinary surgeon 6 (Male, partner 50-65)** - **Veterinary surgeon 7(Male partner, 35-50)** - **Veterinary surgeon 8(Male, partner 35-50)** - **Veterinary surgeon 9(Male partner, 35-50)** - **Veterinary surgeon 10(Male, 35-50)** - **Veterinary surgeon 11(Male, 35-50)** - **Veterinary surgeon 12(Male, 25-35)** - **Veterinary surgeon 13(Male, 25-35)** - **Veterinary surgeon 14 (female, 25-35)** - **Veterinary surgeon 15 (Male, partner 50-65)**   **Veterinary practice 4: South**   - **Veterinary surgeon 16 (Male, partner 35-50)**   **Veterinary surgeons University of Liverpool**   - **Veterinary surgeon/researcher 17(Female, 25-35)** - **Veterinary surgeon/researcher 18 (Male, 25-35)** - **Veterinary surgeon/researcher 19 (Male 45-60)**   **Veterinary consultants**   - **Veterinary surgeon 20 (Male, age 35-45)** - **Veterinary surgeon 21 (Male, age 35-45)** |
| Farmers | - **Farmer 1 midlands (Male, 45-50)** - **Farmer 2 midlands (Male, 45-55)** - **Farmer 3 South (Male, 45-55)** |
| Retailer farmer meetings | **Retailer-Farmer meeting 1**  **Retailer-Farmer meeting 2**  **Retailer-Farmer meeting 3**  **Retailer-Farmer meeting 4**  **Retailer-Farmer meeting 5**  **Retailer-Farmer meeting 6**  **Retailer-Farmer meeting 7**  **Retailer-Famer meeting 8**  **Retailer-Farmer meeting 9**  **Retailer-Farmer meeting 10** |
| Retailer focus groups | **Retailer focus group 1**   - **Farmer (Male, 45-50)** - **Farmer (Male, 45-55)** - **Farmer (Male, 50-65)** - **Farmer (Female, 45-55)** - **Farmer (Female, 45-5)**   **Retailer focus group 2**   - **Farmer (Male, 45-50)** - **Herdsman (Male, 25-35)** - **Farmer (Male, 50-65)** - **Farmer (Female, 45-55)** - **Farmer (Female, 50-65)** |
|  |  |
